# Threshold Crossover of Transmitted Larmor Time in Trapezoidal Barriers: A Quantum-Clock Analysis

**DOI:** 10.3390/e28080864

**Published:** 2026-08-01

**Authors:** Zhuo Li, Zhi Xiao, Tengfei Li

**Affiliations:** 1 School of Mathematics and Physics, North China Electric Power University, Beijing 102206, China; litengfei4944@ncepu.edu.cn; 2 Hebei Key Laboratory of Physics and Energy Technology, North China Electric Power University, Baoding 071000, China

**Keywords:** quantum tunneling, Larmor clock, quantum clock, tunneling time, trapezoidal barrier, threshold crossover, quantum measurement

## Abstract

The Larmor clock extracts time information from quantum scattering through a weak spin-dependent phase readout. We apply this formulation to the transmitted Larmor time in trapezoidal barriers and examine its geometry-dependent threshold response. The main quantity is the dimensionless peak coordinate $x_{p}=E_{p}/V_{0}$, where $E_{p}$ is the energy of the dominant transmitted-time peak. For a right trapezoidal barrier consisting of a linear ramp and a flat segment, the peak moves from the classically forbidden side ($x_{p}<1$) to the allowed side ($x_{p}>1$) as the flat part becomes more important. The crossover is characterized by $r_{c}=\left(l-a\right)/a$, defined through $x_{p}=1$. For a=2 nm and $V_{0}=2\;\mu \mathrm{eV}$, we obtain $r_{c}≃0.428$. A symmetric triangle–rectangle–triangle barrier shows a corresponding crossover at $s_{c}=\left(d-a\right)/\left(2a\right)≃0.220$. These results demonstrate that the strongest transmitted-channel clock response is controlled by the spatial distribution of the sloped matching regions and the flat phase-accumulating region. The peak coordinate therefore provides a phase-sensitive marker of how this response shifts across the nominal threshold as the barrier geometry is varied.

## 1. Introduction

Quantum tunneling is one of the most basic consequences of wave mechanics. A related question, already raised in early studies of wave-packet scattering, is how much time a particle spends in a barrier region. Because time is not represented by a standard self-adjoint operator in non-relativistic quantum mechanics, several operational time scales have been proposed, including the Wigner phase time, dwell time, Büttiker–Landauer time, Pollak–Miller time, and Larmor time [1,2,3,4,5,6,7,8,9,10,11,12].

Among these definitions, the Larmor-clock construction is especially useful from the viewpoint of quantum measurement. A weak magnetic field is applied only in the spatial region whose traversal time is to be probed. The spin degree of freedom then acts as an internal clock, and the spin-dependent scattering phase encodes a time scale. In this sense, the Larmor time can be interpreted as information extracted from a weakly perturbed and postselected scattering process rather than as a classical time of flight assigned to an individual trajectory [9,10,13,14,15,16]. The measured signal is therefore sensitive not only to the magnitude of the transmission coefficient but also to the phase accumulated at the interfaces and inside the barrier [11,17,18,19].

Most discussions of one-dimensional barriers use rectangular or linear potentials as limiting models. A rectangular barrier has two abrupt interfaces and a flat internal region, while a triangular barrier has a single linearly graded region. A trapezoidal barrier provides a simple interpolation between these two limits: it contains both a graded ramp and a flat plateau. This makes it a convenient model for asking a more specific clock-readout question: when the relative proportion of the ramp and the plateau is changed, does the dominant transmitted Larmor-time peak remain on the tunneling side of the classical threshold, or can it cross to the allowed side?

Trapezoidal-barrier Larmor times were recently studied for three configurations: the left-ramp/right-flat one-sided trapezoidal barrier (t-RTB), the reversed left-flat/right-ramp barrier (r-RTB), and the triangle–rectangle–triangle (TRT) barrier [20]. In the following, RTB denotes the one-sided trapezoidal-barrier geometry. The previous study emphasized the dependence on barrier height and width and the relations between alternative clock definitions. The present work addresses a different and more specific question: the migration of the dominant transmitted Larmor-time peak across the classical threshold. We introduce the normalized peak coordinate $x_{p}=E_{p}/V_{0}$, determine the critical geometric ratios at which $x_{p}=1$, and interpret the crossover as a change in the maximum phase response of the postselected transmitted channel.

The present paper addresses this question through a peak-position analysis. The central observable is

(1) $$x_{p}=\frac{E_{p}}{V_{0}},$$

where $E_{p}$ denotes the energy at which the transmitted Larmor time has its dominant peak. The values $x_{p}<1$, $x_{p}=1$, and $x_{p}>1$ respectively correspond to a peak in the classically forbidden region, at the classical threshold, and in the classically allowed region. This formulation deliberately avoids using the near-threshold singular decomposition as the main argument. Instead, it uses the peak position itself as an indicator of how the quantum-clock response changes when the barrier geometry is varied.

From the perspective of information extraction in quantum scattering, the key point is that the transmitted Larmor time is obtained from the spin-dependent phase difference of the transmitted channel. The barrier geometry changes the phase matching at the sloped and flat parts of the potential, and consequently changes the incident energy at which the clock response is maximal. We therefore interpret the critical geometry as the point at which the maximum transmitted-channel clock response moves across the threshold $E=V_{0}$.

## 2. Larmor-Clock Formulation and Barrier Profiles

We consider a particle of mass *m* moving along the *z* direction through a one-dimensional potential barrier V(z). For every transmitted-time calculation presented in this work, a weak magnetic field $B_{0}$ is confined to the potential-barrier region. Thus, the left boundary of the clock region coincides with the left boundary of the barrier; in the notation of the original schematic, this corresponds to b=0. For the longitudinal clock geometry used here, the Hamiltonian can be written as

(2) $$\hat{H}=\frac{{\hat{p}}_{z}^{2}}{2m}+V\left(z\right)-\hat{\mu }\cdot B\;\Omega \left(z\right),$$

where Ω(z)=1 inside the clock region and Ω(z)=0 outside it. The magnetic moment is written as

(3) $$\hat{\mu }=\frac{\hbar \gamma }{2}\hat{\sigma },$$

with gyromagnetic ratio γ and Pauli matrix vector $\hat{\sigma }$. In the weak-field limit, the two spin channels experience slightly shifted potentials,

(4) $$V_{\pm }\left(z\right)=V\left(z\right)\mp \frac{\hbar \gamma B_{0}}{2}\;\Omega \left(z\right).$$

Let $t_{\pm }$ and $r_{\pm }$ be the corresponding transmission and reflection amplitudes. The transmitted and reflected Larmor times are defined by the weak-field phase response

(5) τT=limB0→01γB0Imlnt+t−,     τR=limB0→01γB0Imlnr+r−.

For the finite field used numerically, the right-hand sides of Equation (5) are evaluated after continuously unwrapping the relative phase. We also repeated the calculation after halving and doubling $B_{0}$; the peak coordinates and the reported critical ratios were unchanged within the displayed precision, confirming that the numerical results are in the weak-field linear-response regime.

To see why Equation (5) gives the clock reading, suppose that the incident spin is initially polarized perpendicular to the magnetic field,

$$|\chi_{\mathrm{in}}\rangle =\frac{1}{\sqrt{2}}\left(|+\rangle +|-\rangle \right),\;\;\;\;\;{\hat{\sigma }}_{z}|\pm \rangle =\pm |\pm \rangle .$$

After postselection on either the transmitted or reflected channel, the normalized spin state is

$$|\chi_{H}\rangle =\frac{H_{+}|+\rangle +H_{-}|-\rangle }{\sqrt{|H_{+}{|}^{2}+{\left|H_{-}\right|}^{2}}},\;\;\;\;\;H=t\;\mathrm{or}\;r.$$

Writing $H_{\pm }=\left|H_{\pm }\right|e^{i\phi_{\pm }}$, the relative phase of the two spin components is

δϕH=ϕ+−ϕ−=ImlnH+H−.

This relative phase is the azimuthal Larmor-clock readout. Since the Larmor angular frequency is $\omega_{L}=\gamma B_{0}$, division by $\omega_{L}$ gives Equation (5). By contrast, a modulus imbalance $|H_{+}\left|\neq \right|H_{-}|$ produces a small spin-filtering or alignment component along the magnetic field; it is distinct from the transverse precession used to define the Larmor time. The weak field used in the calculation therefore acts as a clock and does not alter the unperturbed scattering profile appreciably.

For the numerical calculations, we use the model parameters

(6) $$m=1.45718689977\times 10^{-25}\;\mathrm{kg},$$

and

(7) $$\gamma =1.83247\times 10^{8}\;s^{-1}T^{-1}.$$

These parameters determine the kinetic and spin-dependent clock scales used throughout the numerical calculations. The clock field is fixed at

(8) $$B_{0}=1.0\times 10^{-7}\;T=1\;\mathrm{mG}.$$

Energies are quoted in μeV and lengths in nm. With this choice, the spin-dependent energy shift $\hbar \gamma B_{0}/2$ is much smaller than the barrier heights considered below, so the calculation remains in the weak-clock regime. For the representative value $V_{0}=2\;\mu \mathrm{eV}$, the dimensionless clock perturbation is

(9) $$\epsilon_{B}=\frac{\hbar \gamma B_{0}/2}{V_{0}}≃3.02\times 10^{-9},$$

which confirms that the magnetic field acts as a readout probe rather than as a relevant modification of the barrier. The phase readout of the transmitted channel is

(10) ΔϕT(E,g)=Imlnt+(E,g)t−(E,g),     τT(E,g)=ΔϕT(E,g)γB0,

where *g* denotes the chosen geometrical ratio. This relation is used below to interpret the peak migration as a change in the incident energy at which the spin-dependent phase response is maximal. Here the term “information” refers specifically to the phase information contained in the spin-dependent transmission amplitudes. We do not introduce an additional entropy functional; instead, the weak Larmor clock converts the small spin splitting into the measurable phase difference $\Delta \phi_{T}$, from which the transmitted time scale is extracted.

**Figure 1:**
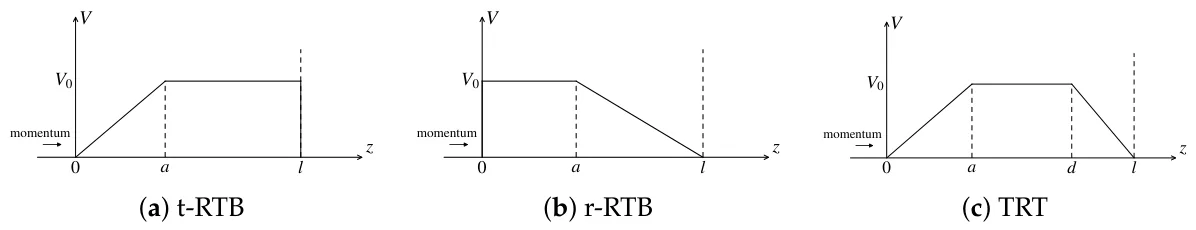
Trapezoidal barrier profiles considered in the calculation. The t-RTB and r-RTB profiles contain a linear ramp and a flat segment in opposite spatial orders, while the TRT profile contains two linear ramps and a central flat segment. In all transmitted-time calculations, the weak clock field is confined to the barrier region 0<z<l.

The three trapezoidal profiles used in this work are shown in Figure 1. The main calculation focuses on the t-RTB profile, which has a left ramp and a right flat segment:

(11) $$V_{t}\left(z\right)=\left\{\begin{array}{cc} V_{0}z/a, & 0<z<a,\\ V_{0}, & a<z<l,\\ 0, & \mathrm{otherwise}. \end{array}\right.$$

For comparison, we also use the oppositely ordered trapezoidal profile r-RTB,

(12) $$V_{r}\left(z\right)=\left\{\begin{array}{cc} V_{0}, & 0<z<a,\\ V_{0}\left(l-z\right)/\left(l-a\right), & a<z<l,\\ 0, & \mathrm{otherwise}, \end{array}\right.$$

and a triangle–rectangle–triangle profile,

(13) $$V_{\mathrm{TRT}}\left(z\right)=\left\{\begin{array}{cc} V_{0}z/a, & 0<z<a,\\ V_{0}, & a<z<d,\\ V_{0}\left(l-z\right)/\left(l-d\right), & d<z<l,\\ 0, & \mathrm{otherwise}. \end{array}\right.$$

The linearly varying regions are solved in terms of Airy functions, and the flat regions are represented by plane waves or exponential waves depending on the energy. Matching the wave function and its derivative at each interface gives the transfer matrix $M_{\pm }$ for each spin channel. With the convention used in the calculation,

(14) $$t_{\pm }=\frac{\det M_{\pm }}{{\left(M_{\pm }\right)}_{22}},\;\;\;\;\;r_{\pm }=-\frac{{\left(M_{\pm }\right)}_{21}}{{\left(M_{\pm }\right)}_{22}}.$$

This standard transfer-matrix construction keeps the Airy matching at the graded segments and the ordinary interface matching at the flat segments in a single framework [21]. The explicit spin-channel matrices for all barrier profiles and a derivation of Equation (14) are given in Appendix A.

## 3. Peak Coordinate as a Crossover Indicator

For a barrier with height $V_{0}$, the classical threshold is $E=V_{0}$. Instead of comparing the peak position in wave-number space alone, it is more transparent to use the dimensionless coordinate $x_{p}=E_{p}/V_{0}$. The transmitted Larmor-time peak is classified as follows:

(15) $$\begin{array}{ccc} x_{p}<1, & \mathrm{forbidden-side}\;\mathrm{peak}, & E_{p}<V_{0},\\ x_{p}=1, & \mathrm{threshold}\;\mathrm{peak}, & E_{p}=V_{0},\\ x_{p}>1, & \mathrm{allowed-side}\;\mathrm{peak}, & E_{p}>V_{0}. \end{array}$$

This definition is useful because it separates the geometric migration of the peak from the trivial movement of the threshold wave number,

(16) $$k_{0}=\frac{\sqrt{2mV_{0}}}{\hbar }.$$

**Figure 2:**
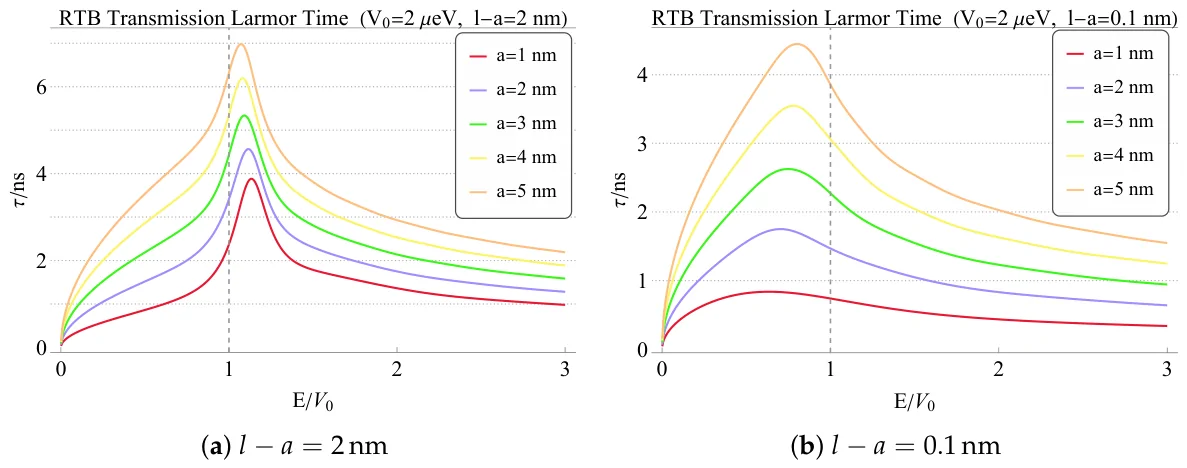
Transmitted Larmor time of the t-RTB as a function of $E/V_{0}$ for two rectangular-segment widths. The vertical dashed line indicates the classical threshold $E=V_{0}$. A longer flat segment drives the peak toward the threshold and into the allowed side, whereas a very short flat segment leaves the peak on the forbidden side.

Figure 2 shows two representative t-RTB calculations at $V_{0}=2\;\mu \mathrm{eV}$. In both panels the vertical dashed line marks $E/V_{0}=1$. When the rectangular segment is relatively long, as in Figure 2a, the dominant peak is located near the threshold and slightly enters the allowed side. When the rectangular segment is reduced to 0.1 nm, as in Figure 2b, the peak remains on the forbidden side. These two cases already show that the peak position is not fixed by the barrier height alone; it is controlled by the relative amount of linearly graded and flat potential inside the barrier.

The geometric control can be quantified by introducing

(17) $$r=\frac{l-a}{a},$$

which measures the width of the flat rectangular segment relative to the linear ramp. Small *r* corresponds to a triangular-like barrier; large *r* corresponds to a more rectangular-like barrier. The crossover point $r_{c}$ is defined by

(18) $$x_{p}\left(r_{c}\right)=1.$$

This definition does not assume a particular analytic form for the Larmor time. It only uses the numerically extracted location of the dominant transmitted-time peak.

**Figure 3:**
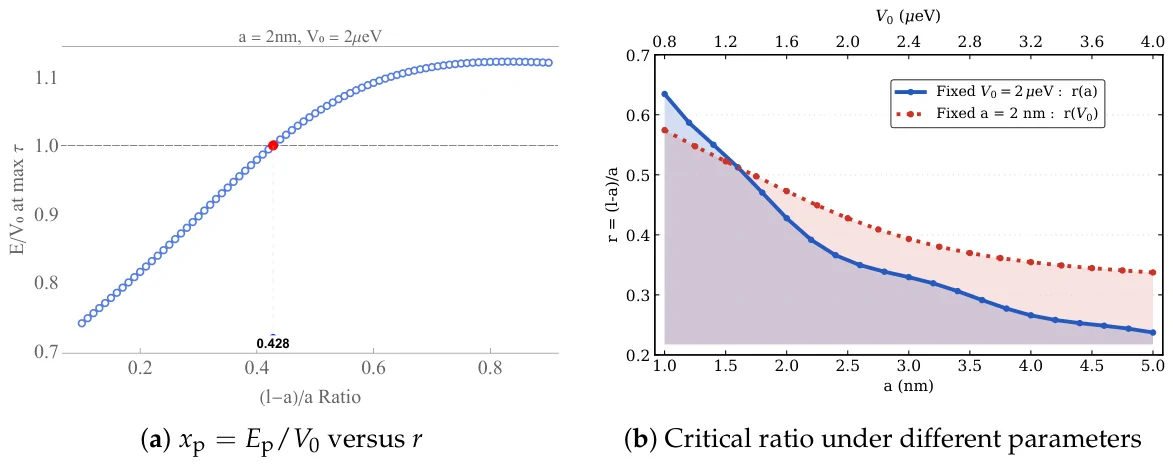
Critical geometric ratio obtained from the condition $E_{p}/V_{0}=1$. (**a**) For a=2 nm and $V_{0}=2\;\mu \mathrm{eV}$, the transmitted Larmor-time peak crosses the classical threshold at $r_{c}≃0.428$. The horizontal dashed line indicates $E_{p}/V_{0}=1$, the vertical dashed line marks $r_{c}$, and the red dot identifies their intersection $\left(r_{c},1\right)$. (**b**) The critical ratio changes when either the ramp width *a* or the barrier height $V_{0}$ is varied. The shaded domains denote parameter regions in which the peak remains on the classically forbidden side.

For reproducibility, the peak coordinate is obtained by a fixed numerical extraction rule. The detailed procedure is given in Appendix B, and the complete critical-ratio data used to support Figure 3b are listed in Appendix C.

## 4. Geometry-Driven Crossing of the Classical Threshold

Figure 3a gives $x_{p}$ as a function of *r* for the reference parameters a=2 nm and $V_{0}=2\;\mu \mathrm{eV}$. The curve increases with *r* and crosses the line $x_{p}=1$. The extracted critical ratio is

(19) $$r_{c}≃0.428.$$

Thus, for this reference barrier, the peak is in the classically forbidden region for $r<r_{c}$, at the threshold for $r=r_{c}$, and in the classically allowed region for $r>r_{c}$.

The second panel of Figure 3, together with the representative values in Table 1 and the full data in Appendix C, shows how $r_{c}$ changes with the ramp coordinate and the barrier height. Increasing either *a* or $V_{0}$ lowers the critical ratio. Equivalently, when the effective strength of the barrier is increased, a smaller fraction of the flat region is sufficient to move the peak to the threshold or beyond it.

A useful way to describe the mechanism is to separate the interface matching from the internal propagation. For a trapezoidal barrier with a flat part, the transmission amplitude may be written schematically in the multiple-reflection form

(20) $$T\left(E\right)=\frac{T_{L}\left(E\right)T_{R}\left(E\right)e^{i\phi (E)}}{1-R_{L}\left(E\right)R_{R}\left(E\right)e^{2i\phi (E)}},$$

where $T_{L}$ and $T_{R}$ describe the left and right matching factors, $R_{L}$ and $R_{R}$ describe internal reflection factors, and ϕ denotes the propagation phase accumulated across the flat segment. The actual calculation uses the full Airy-function transfer matrix, but Equation (20) clarifies why both parts of the barrier matter. The ramp changes the coupling and phase matching into the internal region, while the flat part supplies propagation and repeated internal reflections. The peak of $\tau_{T}$ occurs where the transmitted-channel relative phase is most responsive to the weak local clock perturbation.

It is important, however, not to interpret Equation (5) as assigning a unique trajectory-resolved classical residence time to every transmitted particle. In the weak-field regime, the Larmor clock probes the response of a postselected scattering amplitude to a small spin-dependent perturbation localized in the clock region. Quantum alternatives associated with different durations contribute coherently rather than through an ordinary classical probability distribution [16]. The peak studied here therefore identifies the incident energy at which the transmitted-channel clock phase is most sensitive to that local perturbation.

Accordingly, the threshold crossover does not mean that an individual particle suddenly changes from a definite tunneling trajectory to a definite above-barrier trajectory. It means that the energy of maximum transmitted-channel clock response moves from $E<V_{0}$ to $E>V_{0}$ as the relative importance of ramp matching and plateau propagation is varied. The critical ratio $r_{c}$ is the geometry at which this maximum response passes through the nominal threshold.

This observation gives a practical interpretation of $x_{p}$. For triangular-like barriers the maximum transmitted-channel phase response occurs below the classical threshold, and $x_{p}$ remains below unity. For rectangular-like barriers the flat internal region concentrates this response near the classical threshold, and $x_{p}$ moves to unity or above it. The change of $x_{p}$ therefore provides a direct diagnostic for the transition between triangular-like and rectangular-like Larmor-time responses.

## 5. TRT Comparison as a Symmetric Reference

The threshold crossover is not limited to the one-sided RTB geometry. To test this point, we also consider the symmetric TRT barrier in Figure 1c as a reference structure. For the comparison presented in this section, we impose

(21) l−d=a,

so that the two linear ramps have the same width *a*. The TRT barrier is used here only as a reference geometry; its role is to test whether the threshold crossover persists when the sloped matching regions are distributed symmetrically on both sides of the flat region, rather than to introduce a separate optimization problem. The central plateau has width d−a, and its natural dimensionless parameter is

(22) $$s=\frac{d-a}{2a},$$

which measures the central flat region relative to the two linear ramps. The use of *s* distinguishes the TRT parameter from the RTB ratio r=(l−a)/a.

**Figure 4:**
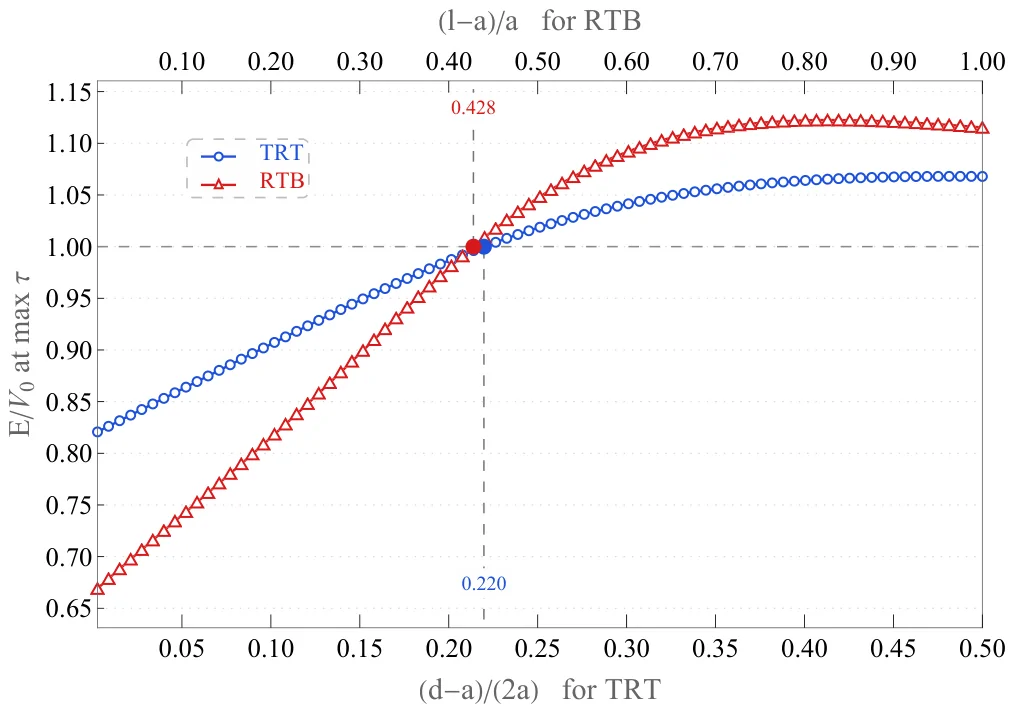
Comparison of threshold crossover in RTB and TRT geometries. The upper horizontal axis refers to the RTB ratio r=(l−a)/a, while the lower horizontal axis refers to the TRT parameter s=(d−a)/(2a). The horizontal dash-dotted line indicates the classical threshold $E/V_{0}=1$. The red and blue dots mark the RTB and TRT threshold-crossing points, respectively, and the vertical dashed lines indicate the corresponding critical ratios. The reference values are $r_{c}^{\mathrm{RTB}}≃0.428$ and $s_{c}^{\mathrm{TRT}}≃0.220$.

Figure 4 shows that the TRT barrier also exhibits a threshold crossover of the transmitted Larmor-time peak. The crossing occurs at $s_{c}^{\mathrm{TRT}}≃0.220$, compared with $r_{c}^{\mathrm{RTB}}≃0.428$ for the one-sided RTB barrier. Because *r* and *s* use different geometric normalizations, their numerical values should not be read as a direct comparison of equal physical lengths. The difference reflects that the flat phase-accumulating region in the TRT geometry is placed between two sloped matching regions. The clock signal is therefore affected by a more symmetric distribution of matching and propagation phases. The comparison shows that the crossover is not an isolated feature of a single asymmetric profile but a more general manifestation of how the Larmor-clock readout responds to the spatial distribution of sloped and flat barrier regions.

## 6. Comparison with Limiting Barriers

**Figure 5:**
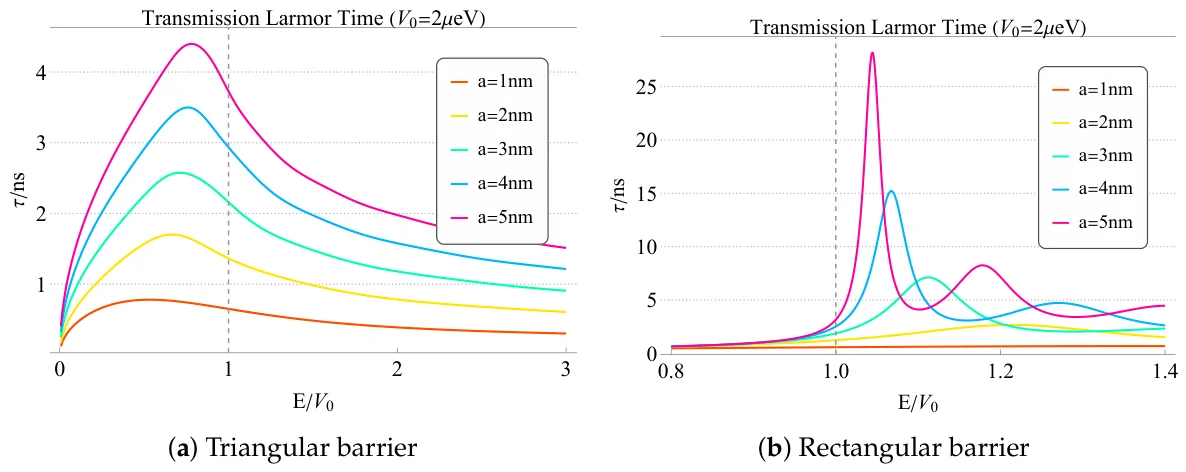
Reference transmitted Larmor-time profiles for triangular and rectangular barriers plotted against $E/V_{0}$. The vertical dashed lines indicate the classical threshold $E/V_{0}=1$. The difference between the broad triangular response and the sharp rectangular threshold response explains why the trapezoidal peak position can move across $E/V_{0}=1$ as the flat part is enlarged.

The above interpretation is supported by the limiting triangular and rectangular cases. Figure 5 compares the transmitted Larmor time as a function of $E/V_{0}$ for pure triangular and pure rectangular barriers. The triangular barrier gives broad peaks located mainly on the forbidden side in the displayed parameter range. The rectangular barrier produces a much sharper threshold-localized structure. The t-RTB crossover in Figure 3 can therefore be viewed as a continuous migration between these two limiting behaviors.

**Figure 6:**
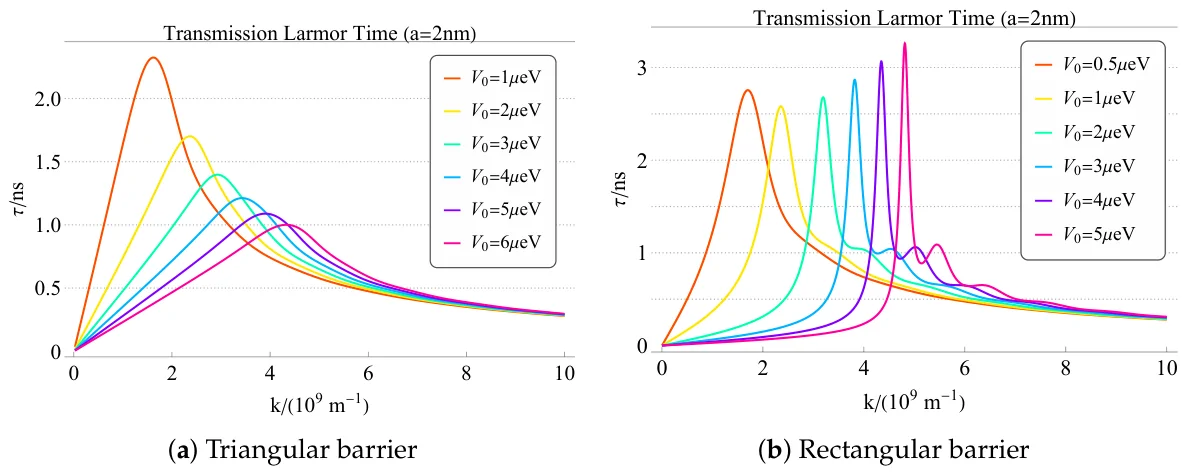
Transmitted Larmor time as a function of wave number for triangular and rectangular reference barriers under varying barrier height. The two limiting profiles display distinct peak-evolution patterns, which are interpolated by the trapezoidal barrier.

A similar contrast appears when the barrier height is varied at fixed geometric scale, as shown in Figure 6. The triangular case exhibits a smoother movement and broadening of the peak, while the rectangular case develops a more pronounced near-threshold maximum. These comparisons show that the peak-position crossover is not merely a consequence of rescaling the energy by $V_{0}$. It reflects the different phase-matching roles of a smooth ramp and an abrupt plateau.

## 7. Conclusions

We have studied the threshold crossover of the transmitted Larmor time in trapezoidal quantum barriers from a quantum-clock perspective. The main quantity is the dimensionless peak coordinate $x_{p}=E_{p}/V_{0}$, which identifies whether the dominant transmitted-time peak lies in the classically forbidden region, at the threshold, or in the classically allowed region. For a right trapezoidal barrier, increasing the relative size of the flat segment drives $x_{p}$ from below unity to above unity. The critical geometric ratio $r_{c}=\left(l-a\right)/a$ is obtained from $x_{p}=1$, and for the reference case a=2 nm, $V_{0}=2\;\mu \mathrm{eV}$, the refined numerical value is $r_{c}≃0.428$.

The result can be interpreted as a change in the spin-dependent phase response extracted by the weak Larmor clock. The sloped part of the barrier controls matching into the internal region, while the flat part contributes propagation and multiple-reflection effects. The critical ratio marks the point at which these contributions move the maximum transmitted-channel clock response through the classical threshold. This interpretation is supported by the dependence of $r_{c}$ on *a* and $V_{0}$, by the comparison with triangular and rectangular limiting profiles, and by the TRT reference calculation, which gives a corresponding symmetric-geometry crossover at $s_{c}≃0.220$.

Consistent with the operational interpretation of a weak Larmor clock, the reported quantities characterize the response of a postselected transmission amplitude to a weak local perturbation and are not interpreted as unique trajectory-resolved durations. The transmitted Larmor-time peak coordinate is therefore a phase-sensitive descriptor of the threshold response rather than a classical traversal-time assignment. It provides a practical marker for comparing threshold-sensitive Larmor-clock responses in different one-dimensional barrier geometries.

## Figures and Tables

**Table 1:** Representative critical geometric ratios extracted from $x_{p}\left(r_{c}\right)=1$. Only selected values are retained in the main text; the complete numerical data are given in Appendix C.

Fixed $V_{0}=2\;\mu \mathrm{eV}$		Fixed a=2 nm	
**a (nm)**	** $r_{c}$ **	**$V_{0}$ (μeV)**	** $r_{c}$ **
1.0	0.635	0.8	0.574
2.0	0.428	2.0	0.428
3.0	0.330	3.2	0.355
5.0	0.237	4.0	0.337

## Data Availability

The numerical data supporting the findings of this study are available from the corresponding author upon reasonable request.

## Appendix A. Spin-Channel Transfer Matrices

This appendix presents the explicit spin-channel transfer matrices used in Equation (14). Because the clock field is confined to the potential barrier in all transmitted-time calculations, the free wave number outside the barrier is the same for the two spin channels,

(A1) $$k=\frac{\sqrt{2mE}}{\hbar }.$$

Let σ=±1 label the eigenvalue of the spin projection along the magnetic field and define

(A2) $$\Delta =\frac{\hbar \gamma B_{0}}{2},\;\;\;\;\;q_{\sigma }=\frac{\sqrt{2m(E-V_{0}+\sigma \Delta )}}{\hbar },$$

where the square-root branch is chosen so that Imqσ≥0. Thus $q_{\sigma }$ is purely imaginary below the corresponding spin-channel threshold.

For a constant-potential region, introduce the basis matrix

(A3) $$P\left(p,z\right)=\left(\begin{array}{cc} e^{ipz} & e^{-ipz}\\ ip\;e^{ipz} & -ip\;e^{-ipz} \end{array}\right),$$

which maps the coefficients of the two basis waves to ${\left(\psi ,\psi^{\prime }\right)}^{T}$ at position *z*.

For a rising linear segment V(z)=αz, the solutions are expressed in terms of the Airy functions of the first and second kinds, denoted by Ai and Bi, respectively. Define

(A4) $$\lambda_{\alpha }={\left(\frac{2m\alpha }{\hbar^{2}}\right)}^{1/3},\;\;\;\;\;\xi_{\alpha \sigma }\left(z\right)=\lambda_{\alpha }\left[z-\frac{E+\sigma \Delta }{\alpha }\right],$$

and

(A5) $$A_{\alpha \sigma }\left(z\right)=\left(\begin{array}{cc} \mathrm{Ai}\;\left(\xi_{\alpha \sigma }\left(z\right)\right) & \mathrm{Bi}\;\left(\xi_{\alpha \sigma }\left(z\right)\right)\\ \lambda_{\alpha }{\mathrm{Ai}}^{\prime }\;\left(\xi_{\alpha \sigma }\left(z\right)\right) & \lambda_{\alpha }{\mathrm{Bi}}^{\prime }\;\left(\xi_{\alpha \sigma }\left(z\right)\right) \end{array}\right).$$

For a descending linear segment V(z)=β(l−z), use the local coordinate x=l−z and define

(A6) $$\lambda_{\beta }={\left(\frac{2m\beta }{\hbar^{2}}\right)}^{1/3},\;\eta_{\beta \sigma }\left(z\right)=\lambda_{\beta }\left[l-z-\frac{E+\sigma \Delta }{\beta }\right].$$

The corresponding basis matrix is

(A7) $$D_{\beta \sigma }\left(z\right)=\left(\begin{array}{cc} \mathrm{Ai}\;\left(\eta_{\beta \sigma }\left(z\right)\right) & \mathrm{Bi}\;\left(\eta_{\beta \sigma }\left(z\right)\right)\\ -\lambda_{\beta }{\mathrm{Ai}}^{\prime }\;\left(\eta_{\beta \sigma }\left(z\right)\right) & -\lambda_{\beta }{\mathrm{Bi}}^{\prime }\;\left(\eta_{\beta \sigma }\left(z\right)\right) \end{array}\right).$$

The minus signs in the second row follow from $d\eta_{\beta \sigma }/dz=-\lambda_{\beta }$.

We use the left-to-right convention

(A8) $$\left(\begin{array}{c} B_{1}\\ B_{2} \end{array}\right)=M_{\sigma }\left(\begin{array}{c} A_{1}\\ A_{2} \end{array}\right),$$

where $\left(A_{1},A_{2}\right)$ and $\left(B_{1},B_{2}\right)$ are the right- and left-moving asymptotic amplitudes on the left and right sides of the barrier, respectively. The matrices for the profiles used in the manuscript are listed below.

### Appendix A.1. Triangular and Rectangular Limits

For a rising triangular barrier over 0<z<L, with $\alpha =V_{0}/L$,

(A9) $$M_{\sigma }^{\mathrm{tri}}=P^{-1}\left(k,L\right)A_{\alpha \sigma }\left(L\right)A_{\alpha \sigma }^{-1}\left(0\right)P\left(k,0\right).$$

For a rectangular barrier over 0<z<L,

(A10) $$M_{\sigma }^{\mathrm{rect}}=P^{-1}\left(k,L\right)P\left(q_{\sigma },L\right)P^{-1}\left(q_{\sigma },0\right)P\left(k,0\right).$$

### Appendix A.2. t-RTB, r-RTB, and TRT Barriers

For the t-RTB potential in Equation (11), with $\alpha =V_{0}/a$,

(A11) $$M_{\sigma }^{t-\mathrm{RTB}}=P^{-1}\left(k,l\right)P\left(q_{\sigma },l\right)P^{-1}\left(q_{\sigma },a\right)A_{\alpha \sigma }\left(a\right)A_{\alpha \sigma }^{-1}\left(0\right)P\left(k,0\right).$$

For the r-RTB potential in Equation (12), with $\beta =V_{0}/\left(l-a\right)$,

(A12) $$M_{\sigma }^{r-\mathrm{RTB}}=P^{-1}\left(k,l\right)D_{\beta \sigma }\left(l\right)D_{\beta \sigma }^{-1}\left(a\right)P\left(q_{\sigma },a\right)P^{-1}\left(q_{\sigma },0\right)P\left(k,0\right).$$

For the TRT potential in Equation (13), with $\alpha =V_{0}/a$ and $\beta =V_{0}/\left(l-d\right)$,

(A13) $$\begin{array}{cc} M_{\sigma }^{\mathrm{TRT}}= & P^{-1}\left(k,l\right)D_{\beta \sigma }\left(l\right)D_{\beta \sigma }^{-1}\left(d\right)P\left(q_{\sigma },d\right)P^{-1}\left(q_{\sigma },a\right)\\ & \times \;A_{\alpha \sigma }\left(a\right)A_{\alpha \sigma }^{-1}\left(0\right)P\left(k,0\right). \end{array}$$

For the symmetric TRT scans in Section 5, l−d=a, and hence β=α. The matrices in the main text are $M_{+}=M_{\sigma =+1}$ and $M_{-}=M_{\sigma =-1}$.

At an exact spin-channel threshold $q_{\sigma }=0$, the columns of $P(q_{\sigma },z)$ become linearly dependent. This is only a singularity of the exponential basis, not of the scattering solution. The transfer matrix is evaluated through the continuous limit $q_{\sigma }\to 0$ or, equivalently, by using the regular threshold solution $\psi \left(z\right)=C_{1}+C_{2}z$ with

(A14) $$P_{0}\left(z\right)=\left(\begin{array}{cc} 1 & z\\ 0 & 1 \end{array}\right).$$

For incidence from the left, $A_{1}=1$, $A_{2}=r_{\sigma }$, $B_{1}=t_{\sigma }$, and $B_{2}=0$. Equation (A8) therefore becomes

(A15) $$\left(\begin{array}{c} t_{\sigma }\\ 0 \end{array}\right)=\left(\begin{array}{cc} {\left(M_{\sigma }\right)}_{11} & {\left(M_{\sigma }\right)}_{12}\\ {\left(M_{\sigma }\right)}_{21} & {\left(M_{\sigma }\right)}_{22} \end{array}\right)\left(\begin{array}{c} 1\\ r_{\sigma } \end{array}\right).$$

The second row gives

(A16) $$r_{\sigma }=-\frac{{\left(M_{\sigma }\right)}_{21}}{{\left(M_{\sigma }\right)}_{22}},$$

and substitution into the first row gives

(A17) $$t_{\sigma }={\left(M_{\sigma }\right)}_{11}-\frac{{\left(M_{\sigma }\right)}_{12}{\left(M_{\sigma }\right)}_{21}}{{\left(M_{\sigma }\right)}_{22}}=\frac{\det M_{\sigma }}{{\left(M_{\sigma }\right)}_{22}},$$

which proves Equation (14).

## Appendix B. Numerical Extraction of the Peak Coordinate

This appendix summarizes the numerical rule used to extract the peak coordinate and the critical ratio. For each set of geometric and barrier parameters, the relative phase $\arg[t_{+}\left(E\right)/t_{-}\left(E\right)]$ is continuously unwrapped before the transmitted Larmor time is evaluated. Unless otherwise stated, $\tau_{T}$ is sampled in the dimensionless energy window

(A18) $$10^{-3}\leq E/V_{0}\leq 3.$$

The dominant peak is defined as the largest valid local maximum of $\tau_{T}\left(E\right)$ in this window. After the maximum grid point is found, its position is refined by a local quadratic interpolation using the peak point and its two nearest neighboring grid points. This gives the numerical estimate of $x_{p}=E_{p}/V_{0}$ for a fixed geometry.

For the t-RTB geometry, the flat-to-ramp ratio is

(A19) $$r=\frac{l-a}{a}.$$

The function $x_{p}\left(r\right)$ is obtained by repeating the peak extraction over a sequence of *r* values. A smooth interpolation of $x_{p}\left(r\right)$ is then used to find the threshold-crossing point,

(A20) $$x_{p}\left(r_{c}\right)=1.$$

The same extraction rule is used for the representative values in Table 1, the complete data in Appendix C, and the curves in Figure 3. This procedure is intended to define a reproducible numerical indicator of peak migration; it is not an additional analytic approximation to the Larmor time.

## Appendix C. Complete Critical-Ratio Data

The full numerical values used for the parameter-dependence plot are listed in Table A1 and Table A2. The common reference point a=2 nm and $V_{0}=2\;\mu \mathrm{eV}$ is reported as the refined value $r_{c}=0.42774$, corresponding to $r_{c}≃0.428$ in the main text. The tabulated values are the direct output of the same numerical extraction procedure used for Figure 3.

**Table A1:** Complete values of $r_{c}$ for fixed $V_{0}=2\;\mu \mathrm{eV}$ and varying ramp width *a*.

*a* (nm)	$r_{c}$	*a* (nm)	$r_{c}$	*a* (nm)	$r_{c}$
1.0	0.63465	2.4	0.36614	3.8	0.27723
1.2	0.58689	2.6	0.34961	4.0	0.26599
1.4	0.55010	2.8	0.33868	4.2	0.25822
1.6	0.51252	3.0	0.32959	4.4	0.25298
1.8	0.47035	3.2	0.31929	4.6	0.24868
2.0	0.42774	3.4	0.30628	4.8	0.24378
2.2	0.39180	3.6	0.29140	5.0	0.23735

**Table A2:** Complete values of $r_{c}$ for fixed a=2 nm and varying barrier height $V_{0}$.

$V_{0}$ (μeV)	$r_{c}$	$V_{0}$ (μeV)	$r_{c}$
0.8	0.57447	2.6	0.38023
1.0	0.54755	2.8	0.36973
1.2	0.52251	3.0	0.36132
1.4	0.49752	3.2	0.35457
1.6	0.47278	3.4	0.34911
1.8	0.44920	3.6	0.34460
2.0	0.42774	3.8	0.34076
2.2	0.40901	4.0	0.33736
2.4	0.39322	–	–
